# Gene Repertoire Evolution of *Streptococcus pyogenes* Inferred from Phylogenomic Analysis with *Streptococcus canis* and *Streptococcus dysgalactiae*

**DOI:** 10.1371/journal.pone.0037607

**Published:** 2012-05-30

**Authors:** Tristan Lefébure, Vince P. Richards, Ping Lang, Paulina Pavinski-Bitar, Michael J. Stanhope

**Affiliations:** Department of Population Medicine and Diagnostic Sciences, College of Veterinary Medicine, Cornell University, Ithaca, New York, United States of America; University of South Dakota, United States of America

## Abstract

*Streptococcus pyogenes*, is an important human pathogen classified within the pyogenic group of streptococci, exclusively adapted to the human host. Our goal was to employ a comparative evolutionary approach to better understand the genomic events concomitant with *S. pyogenes* human adaptation. As part of ascertaining these events, we sequenced the genome of one of the potential sister species, the agricultural pathogen *S. canis*, and combined it in a comparative genomics reconciliation analysis with two other closely related species, *Streptococcus dysgalactiae* and *Streptococcus equi*, to determine the genes that were gained and lost during *S. pyogenes* evolution. Genome wide phylogenetic analyses involving 15 *Streptococcus* species provided convincing support for a clade of *S. equi*, *S. pyogenes*, *S. dysgalactiae*, and *S. canis* and suggested that the most likely *S. pyogenes* sister species was *S. dysgalactiae*. The reconciliation analysis identified 113 genes that were gained on the lineage leading to *S. pyogenes*. Almost half (46%) of these gained genes were phage associated and 14 showed significant matches to experimentally verified bacteria virulence factors. Subsequent to the origin of *S. pyogenes*, over half of the phage associated genes were involved in 90 different LGT events, mostly involving different strains of *S. pyogenes*, but with a high proportion involving the horse specific pathogen *S. equi* subsp. *equi*, with the directionality almost exclusively (86%) in the *S. pyogenes* to *S. equi* direction. *Streptococcus agalactiae* appears to have played an important role in the evolution of *S. pyogenes* with a high proportion of LGTs originating from this species. Overall the analysis suggests that *S. pyogenes* adaptation to the human host was achieved in part by (i) the integration of new virulence factors (e.g. *speB*, and the *sal* locus) and (ii) the construction of new regulation networks (e.g. *rgg*, and to some extent *speB*).

## Introduction

*Streptococcus pyogenes* is a leading human pathogen responsible for illness ranging from mild skin and respiratory infections (e.g. pharyngitis and impetigo) to life-threatening invasive (e.g. pneumonia, septicemia, streptococcal toxic shock syndrome, necrotizing fasciitis), and post-infection diseases (e.g. acute rheumatic fever, paediatric autoimmune neuropsychiatric disorders). Many different serotypes and strains have been described, with some being linked to particular disease. For example, strains causing necrotizing fasciitis are largely serotype M1 and M3 [1], while M18 is often linked to acute rheumatic fever [2], and M28 to puerperal sepsis [3]. As part of attempts to understand the nature of this diversity, 12 complete and one draft genome have been sequenced (11 complete at the beginning of this study) [2], [4], [5], [6], [7], [8], [9], [10], [11], [12]. The publications associated with these genomes have suggested links between lisogenic phages, and the virulence factors they are carrying, to specific diseases. A long and detailed list of *S. pyogenes* virulence factors is now available (e.g. [13]). Information is now available regarding the genomic repertoire of *S. pyogenes* (e.g. [14], the link between some virulence factors and disease [15], [16], and the history of lateral gene transfer for some of the loci (e.g. [6], [17], [18]. Nonetheless, many of the molecular details related to the adaptive specifics of this organism remain unknown. *S. pyogenes* is classified within the pyogenic group, which is currently composed of 12 species of *Streptococcus* [19], which inhabit various species of mammals (e.g. bovine, dogs, cats, horse, swine, humans). Most species of the pyogenic group are found in a range of different hosts. *S. pyogenes* is unusual in that it is only found in humans.

The putative sister group to *S. pyogenes* is uncertain. There is phylogenetic evidence suggesting it could be *S. canis* [19], or *Streptococcus dysgalactiae* [20]. Whatever the precise evolutionary history, with ribosomal sequence divergence of around 3%, these three taxa are clearly very closely related [19]. *S. canis* colonizes a variety of hosts including dogs, cats, and cows, with few reported human infections [21], [22]. In addition to causing bovine mastitis [23], *S. canis* shares with *S. pyogenes* the potential to cause similar disease, such as respiratory tract infections [24], streptococcal toxic shock syndrome [25], endocarditis [26], and necrotizing fasciitis [25]. *Streptococcus dysgalactiae* includes two subspecies, *Streptococcus dysgalactiae* subsp *dysgalactiae* and *Streptococcus dysgalactiae* subsp *equisimilis*. *S. dysgalactiae* subsp. *equisimilis*, was primarily regarded as a human commensal organism [27] but is now recognized as an increasingly important human pathogen, linked to a spectrum of human diseases including cellulitis, peritonitis, septic arthritis, pneumonia, endocarditis, acute pharyngitis, bacteremia, and toxic shock syndrome [28], which, like *S. canis*, includes several infections similar to those caused by *S. pyogenes*. *S. dysgalactiae* subsp. *dysgalactiae* on the other hand, is strictly an animal pathogen and a major cause of bovine mastitis. Given the overall lack of host specific adaptation of the taxa within the pyogenic group, concomitant with the characteristics of *S. canis* and *S. dysgalactiae*, it is likely that the ancestor to *S. pyogenes* was not a strict human pathogen, if not a human pathogen at all. This suggests that one of the principal factors in the evolution of *S. pyogenes* was its strict adaptation to the human host.

The sequenced *S. pyogenes* genomes have facilitated the identification of many of the molecular features associated with strain and serotype differentiation, but it remains unclear what makes *S. pyogenes* a strict human pathogen compared to many of the host generalists typical of the pyogenic group. An improved understanding of this issue is important in any attempt to develop a broad medical strategy, such as a GAS vaccine (GAS: group A streptococcus). In this study, we describe the genomic features that evolved since the divergence of *S. pyogenes* from its closest relatives, in an attempt to understand the molecular details associated with *S. pyogenes* development as a strict human pathogen. For this purpose we sequenced the *S. canis* genome and combined it in comparative analysis with genome sequence data from the closely related taxa *S. pyogenes*, *S. dysgalactiae*, and *S. equi*. A closely related taxon provides the ability to ascribe to the *S. pyogenes* branch the specific features of *S. pyogenes* evolution. The use of a less closely related taxon as a reference (e.g. one of the publicly available *S. agalactiae* genomes) would yield a less accurate description because it would merge the evolutionary history of several lineages (e.g. *S. canis*, *S. agalactiae*, *S. iniae* and *S. equi*). More specifically, our purpose was to (1) provide a rigorous genome based phylogenetic perspective on identifying the *S. pyogenes* sister group and (2) identify the genes that were gained and lost along the *S. pyogenes* lineage after the divergence of *S. pyogenes* from its closest relatives.

## Materials and Methods

### Genome sequencing and annotation

*Streptococcus canis* strain FSL Z3-227 was isolated in New York State in 1999 from the milk of dairy cows associated with an outbreak of mastitis [23]. Based on results from bacterial culture and ribotyping, a farm cat with chronic sinusitus was the likely source of the outbreak [23]. The *S. canis* genome was sequenced using 454 pyrosequencing [29] on a FLX sequencer. A total of 128,749 single end reads and 140,788 paired-end reads assembled into 91 contigs (>200 bp) and 8 scaffolds, representing an average 23× site coverage. A physical map of the genome was determined by OpGen Technologies, Inc. (Madison, WI) using restriction enzyme BgIII and the optical mapping technique. The order and orientation of the scaffolds was determined by aligning the scaffold on the optical map using Opgen Mapviewer. Small inter and intra-scaffold gaps were closed by PCR and sequenced using Sanger sequencing, while 7 large gaps were amplified with long range PCR and sequenced on the Illumina GA2 sequencer. The Illumina reads were assembled with Velvet [30] using a large range of parameters and the best assembly was selected using the N50 statistic. Genome annotation was done by NCBI Prokaryotic Genomes Automatic Annotation. This pipeline is composed of HMM-based gene prediction methods and employs a sequence similarity-based approach involving comparison of the predicted gene products to the non-redundant protein database, Entrez Protein Clusters, the Conserved Domain Database, and the COGs (Clusters of Orthologous Groups). This Whole Genome Shotgun project has been deposited at DDBJ/EMBL/GenBank under the accession AIDX00000000. The version described in this paper is the first version, AIDX01000000.

### Orthologous genes

All the complete *Streptococcus* genomes available at the time of this study were collected from NCBI (Table 1). Orthologs were delimited using OrthoMCL2 [31], with post-processing as detailed elsewhere [32], [33]. Briefly, reciprocal BLASTp was performed within and between all genome pairs (e-value cut-off = 1E-5). The resulting e-values were then used to build a normalized similarity matrix, which was analyzed using a Markov Cluster algorithm to delineate proteins into clusters containing sets of orthologs and recent paralogs [34]. Proteins were considered recent paralogs if they were more similar to each other than to any protein from another genome. Fragmented protein sequences, such as those that span separate contigs or insertion sequences, can be erroneously categorized as distinct orthologs. To correct for this, clusters containing single proteins were not considered distinct orthologs (rather fragments of the same protein) if they met the following criteria: (i) showed strong homology with another cluster (i.e. could potentially group together to form a single orthologous cluster), (ii) failed to group together because the protein clustering independently, showed no reciprocal BLASTp hit with one of the proteins in the second cluster, (iii) the two proteins showing no reciprocal BLASTp hit originated from the same genome. Proteins that were larger than 30 amino acids and had no BLASTp hit with any other protein were considered strain specific (E-value≤1e-10). Clusters were annotated by merging annotation of sequences from the same cluster. Potential virulence factors were searched via BLASTp against the VFDB database [35] using the longest sequence of each cluster. Clusters were aligned as described elsewhere [36]. Briefly, sequences are translated into proteins, aligned with Probalign [37], backtranslated into DNA, and sites with low posterior probability masked. Clusters with more than 50% of their sites masked are disregarded from any downstream analysis.

**Table 1:** *Streptococcus* genomes used in the study.

Taxa	Locus tag	Species ID	Accession number
*S. agalactiae* 2603	sag_2603	sag1	NC_004116
*S. agalactiae* A909	sag_A909	sag2	NC_004368
*S. agalactiae* NEM316	sag_NEM	sag3	NC_004368
*S. dysgalactiae* subsp. *equisimilis* GGS 124	sde_GGS	sde	NC_012891
*S. equi* subsp. *equi Se*4047	seq_4047	seq	NC_012471
*S. equi* subsp. *zooepidemicus Sz*H70	sez	sez1	NC_012470
*S. equi* subsp. *zooepidemicus* MGCS10565	sez_M	sez2	NC_011134
*S. gordonii* Challis substr CH1	sgo	sgo	NC_009785
*S. mutans*	smu	smu	NC_004350
*S. pneumoniae* TIGR4	spn	spn	NC_003028
*S. pyogenes* M1 GAS	spy_M1	spy1	NC_002737
*S. pyogenes* Manfredo	spy_Man	spy2	NC_009332
*S. pyogenes* MGAS10270	spy_10270	spy3	NC_008022
*S. pyogenes* MGAS10394	spy_10394	spy4	NC_006086
*S. pyogenes* MGAS10750	spy_10750	spy5	NC_008024
*S. pyogenes* MGAS2096	spy_2065	spy6	NC_008023
*S. pyogenes* MGAS315	spy_315	spy7	NC_004070
*S. pyogenes* MGAS5005	spy_5005	spy8	NC_007297
*S. pyogenes* MGAS6180	spy_6180	spy9	NC_007296
*S. pyogenes* MGAS8232	spy_8232	spya	NC_003485
*S. pyogenes* MGAS9429	spy_9429	spyb	NC_008021
*S. pyogenes* NZ131	spy_NZ	spyc	NC_011375
*S. pyogenes* SSI-1	spy_SSI	spyd	NC_004606
*S. sanguinis* SK36	san	san	NC_009009
*S. suis* SC84	ssu	ssu	NC_012924
*S. thermophilus* LMG 18311	sth	sth	NC_006448
*S. uberis* 0140J	sub	sub	NC_012004
*S. canis*	sca	sca	AIDX00000000
*S. gallolyticus* UCN34 uid46061	GALLO	sga	NC_013798
*S. mitis* B6 uid46097	smi	smi	NC_013853

### Gene trees and species tree reconstruction

Gene trees were reconstructed for all the clusters composed of more than 2 sequences using PhyML [38] with a GTR+G+I model of evolution and the SPR tree search heuristic, with 500 pseudo bootstrap replicates. Total evidence (concatenation of the core genes) and core-gene tree consensus approaches were first used to tentatively infer a species tree. Level of concordance between core gene trees was also assessed using Bucky [39] on a reduced data-set: *S. canis*, *S. dysgalactiae*, *S. equi* and two *S. pyogenes* (spy1 and spy2, Table 1). For each gene independently, MrBayes [40] was used to obtain gene tree posterior probabilities, then Bucky takes into account gene tree concordance, providing a revised posterior probability distribution for each gene and estimates the proportion of the core genes for which any given clade is true.

### Reconciliation

To reconstruct the history of genomic events associated with the emergence of the different pyogenic species, we used the reconciliation approach AnGST [41]. Using a species tree, AnGST reconciles a gene tree with the species tree using a generalized parsimony criterion to infer a minimal set of evolutionary events including gene birth, speciation, gene loss, gene duplication and lateral gene transfer. All the clusters composed of more than two sequences were analyzed by AnGST using the three most common gene trees as species tree and default parameters. These three trees involved different relationships for *S. pyogenes*, *S. canis*, and *S. dysgalactiae*: (spy,sde)sca; (spy,sca)sde; (sca,sde)spy. Inference errors due to phylogenetic uncertainty were minimized by incorporating 500 pseudo-bootstrap replicates per gene. The focus of our examination is the *S. pyogenes* branch, and if an evolutionary event (gene gain, loss etc.) for this branch was judged the same irrespective of which of the three topologies was considered, it was evaluated as robust. Despite the fact there is one topology that is more likely (described below), we adopted this conservative approach, since there is, nonetheless, some uncertainty in the sister group relationship to *S. pyogenes* involving these taxa. A significant proportion of the genes gained on the *S. pyogenes* branch were phage associated. Using the tree with the most likely topology - (spy,sde)sca - we further investigated, using this same AnGST approach, the history of these phage associated genes after they were gained on the spy branch.

## Results and Discussion

### Gene trees and species tree

**Figure 1:**
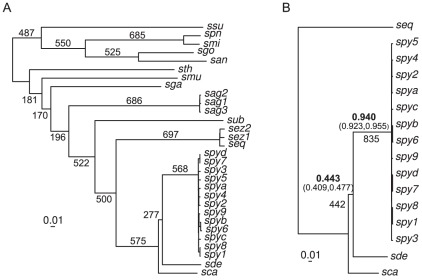
Total evidence trees using the *Streptococcus* one-to-one core genes (701 orthologs, A) and the four species one-to-one core genes (1072 orthologs, B). On the branches are reported the gene-tree support, as well as the genome wide concordance factors (in bold) with their 95% credibility intervals for the four species analysis. For both trees, total evidence topologies are identical to gene-tree majority rule consensus topologies, as well as the primary concordance topology for the four species tree.

**Figure 2:**
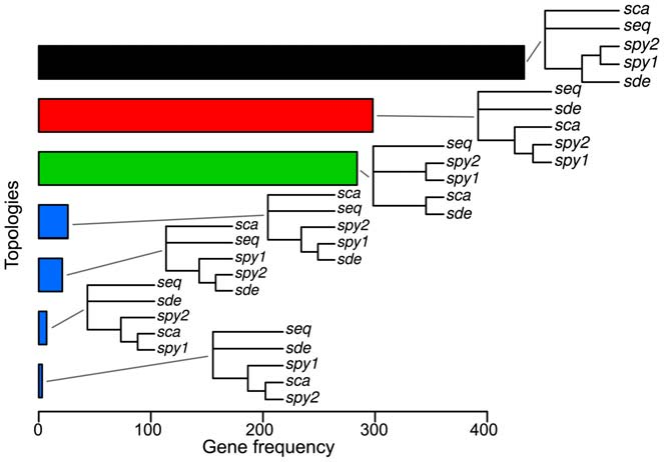
The seven topologies and the number of genes mapped to them, found by the Bayesian gene tree concordance analysis.

**Figure 3:**
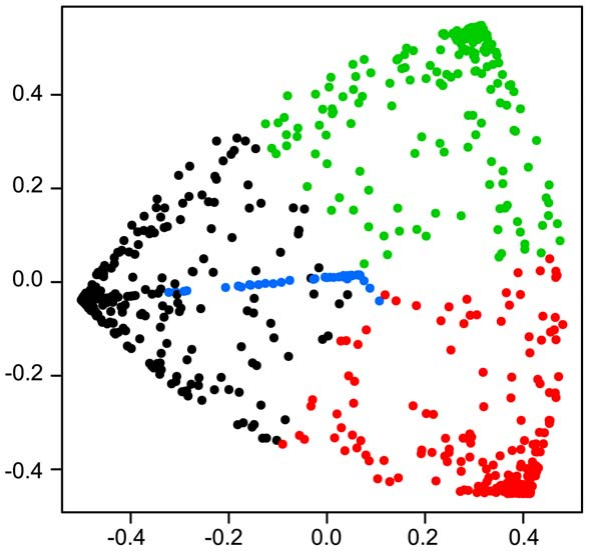
Multidimensional scaling representation of 1072 core genes. Similarity between pairs of genes is measured as the probability that two genes share the same topology. Genes are colored according to their most probable topology, with black referring to topology 1, red is topology 2, and green topology 3; blue refers to other rare topologies (see Table 2 for topology descriptions).

Independent gene tree reconstruction for the *Streptococcus* core genome (701 genes) displayed an overall consensus within the pyogenic clade (*S. agalactiae*, *S. uberis*, *S. equi*, *S. canis*, *S. dysgalactiae* and *S. pyogenes*) with the exception of the relationship between three species: *S. canis*, *S. dysgalactiae* and *S. pyogenes* (Table 2 and Fig. 1). The most common topology was *S. dysgalactiae* and *S. pyogenes* as sister groups (topology 1, 39.5% of the gene trees), followed by the monophyly of *S. canis* and *S. dysgalactiae* (topology 3: 28.7%) and finally the monophyly of *S. canis* and *S. pyogenes* (topology 2: 23%). The majority rule topology was also supported by the total evidence approach (ie. concatenation of the genes prior to phylogenetic reconstruction, Fig. 1). Focusing on a reduced number of taxa (four species) to increase the core-gene sample size (1072 genes), resulted in the same pattern whether using consensus or total evidence approaches (Table 2). Incongruences between gene trees can result from phylogenetic reconstruction problems, incomplete lineage sorting, hidden paralogy and lateral gene transfer (e.g. [42]). If one considers gene tree support for a given topology, whatever the data-set, the gene-tree bootstrap scores did not vary considerably, demonstrating that most of the genes strongly supported one unique topology, suggesting therefore, that reconstruction problems due to lack of phylogenetic signal is not a likely explanation for the incongruence. A Bayesian gene tree concordance analysis was also carried out and whatever the *a priori* discordance parameter α, converged on seven topologies (Fig. 2). The first three topologies are described above, and have associated genome wide concordance factors of 0.443, 0.293 and 0.263, respectively (Table 2). The other four topologies had much fewer genes mapped to them and corresponded to a branching pattern that disrupted *S. pyogenes* monophyly. Very few genes were not mapped to a particular topology (only 56 genes have maximum posterior probability mapping inferior to 0.5), again suggesting that the incongruence between gene trees is unlikely to result from phylogenetic reconstruction problems. When analyzing the posterior probability that two genes share the same topology using a multidimensional scaling representation, we again observed three clear groups of genes corresponding to the three major topologies described above, with very few genes showing alternative patterns (Fig. 3).

**Table 2:** Gene tree support and concordance factors for three concurrent species trees.

	Strep core (701 genes)		4 sp core (1072 genes)		
Topologies	Gene tree	Bootstrap>90%	Gene tree	Bootstrap>90%	Concordance factors
((sde,spy),sca),seq;	277 (39.5%)	262 (37.4%)	442 (41.2%)	430 (40.1%)	0.443 (0.409,0.477)
((sca,spy),sde),seq;	161 (23.0%)	151 (21.5%)	319 (29.8%)	305 (28.5%)	0.293 (0.262,0.326)
((sca,sde),spy),seq;	201 (28.7%)	188 (26.8%)	281 (26.2%)	273 (25.5%)	0.263 (0.233,0.295)

“Strep core” refers to the core genome of the genus *Streptococcus* based on the species included in Table 1. “4 sp core” refers to the core genome based on a consideration of only the 4 species involved in these three topologies. The ‘Gene tree’ column refers to the number of gene trees displaying the specified topology, while the ‘Bootstrap’ column presents the number of genes supporting this bipartition with a bootstrap score greater than 90%. The last column includes the concordance factors with 95% confidence intervals in parentheses.

A large majority of the *Streptococcus* core-genome is composed of single copy genes, so although it is difficult to rule out instances of hidden paralogy, it is unlikely to be the cause of the high frequency of incongruence we observe. Incomplete lineage sorting (also called deep coalescence), is more frequent when divergence time between two speciation events is shorter and population size is larger [42], [43], which both seem plausible in our example. Bacteria have a great propensity to transfer genetic material to other species, and such lateral gene transfer is another likely cause of incongruence among gene trees. Though the issue is not entirely clear-cut, based on total evidence, gene tree consensus and Bayesian concordance analyses, the most likely scenario is that the true species tree has *S. dysgalactiae* and *S. pyogenes* as sister species (topology 1, Table 2), and that a high level of gene transfer between the *S. canis*, *S. dysgalactiae* and *S. pyogenes* lineages resulted in about 50% of the core genes showing an alternative history.

### Genes gained along the *Streptococcus pyogenes* branch

With the goal of identifying the genomic events concomitant with *S. pyogenes* human adaptation, we applied a phylogenomic approach that reconciles observed differences between species and gene phylogenetic trees [41] to a genomic sequence data set of 15 different *Streptococcus* species (Table 1). Including only those genes which were robust to the different topologies defining the *S. pyogenes* sister group, we detected 113 genes that were gained on the branch (lineage) leading to *S. pyogenes* (Table 3). Based on an average number of 1,853 genes among the 13 *S. pyogenes* strains included in the analysis, these gained genes represented 6.1% of an average genome. Of these gained genes, 14 were identified as lateral gene transfers (LGTs) from other species included in the analysis. The remaining 99 genes, within the phylogenetic context of the species tree used in the analysis, were identified as first occurring on the *S. pyogenes* branch. Given that these genes were absent in all the remaining *Streptococcus* species prior to their appearance on the *S. pyogenes* branch, it is possible that these genes were involved in LGT with a bacteria not included in the analysis. We explored this possibility by using BLASTp (e-value cut-off = 1E-5) to search the NCBI NR database for significant sequence matches for each of these genes. A total of 64.6% (64) of the genes matched other species, with 33.3% of these matching a species of *Streptococcus*. Six genes identified as gained on the *S. pyogenes* branch, matched other *Streptococcus* species included in the analysis, the consequence of LGT events involving *S. pyogenes* as donor, subsequent to the original gain. The remaining 29 genes matched only *S. pyogenes*. Consequently, approximately one third of the gained genes had no significant match to any sequenced protein and therefore, these genes either evolved *de-novo* along the *S. pyogenes* branch, or homologous loci have yet to be sequenced. With one exception (*speK*) all of these genes were hypothetical proteins.

**Table 3:** Genes gained on the *Streptococcus pyogenes* branch.

Locus tag	LGT	Gene	Annotated product
19	sag	purC*	phosphoribosylaminoimidazole-succinocarboxamide synthase
61	sag	*adk*	adenylate kinase
110			butyrate-acetoacetate CoA-transferase, beta subunit
155		speG*	pyrogenic exotoxin G precursor
377			putative positive transcriptional regulator
378		*aroE*	shikimate 5-dehydrogenase
386	sga		putative efflux protein
454	sag	*prfB*	peptide chain release factor 2
458	sag		acetoin reductase
461	sag	asnC*	asparaginyl-tRNA synthetase
526		rgpEc*	putative glycosyltransferase
591			5′-nucleotidase
684			putative repressor - phage associated
725		hylP.1*	hyaluronidase - phage associated
799	sag	*prfA*	peptide chain release factor 1
923			putative holin - phage associated
1013		*nadC*	nicotinate-nucleotide pyrophosphorylase
1014			putative integral membrane protein
1101		hylP.3*	hyaluronoglucosaminidase - phage associated
1141		*xis*	putative excisionase - phage associated
1201		yesM*	putative two-component sensor histidine kinase
1205		speK*	streptococcal pyrogenic exotoxin SpeK - phage associated
1221			putative minor capsid protein - phage associated
1223			putative minor capsid protein - phage associated
1225			putative major capsid/head protein - phage associated
1229			putative minor capsid protein - phage associated
1235			putative ABC transporter ATP-binding protein - phage associated
1249			putative single-strand DNA-binding protein - phage associated
1301		speA3*	exotoxin type A precursor - phage associated
1349			putative Cro-like repressor - phage associated
1465		*npx*	NADH peroxidase
1483		*lacC.1*	putative tagatose-6-phosphate kinase
1612	sag	*nupC*	putative nucleoside transporter
1624	sag	*pgk*	phosphoglycerate kinase
1645		salR*	putative response regulator of salavaricin regulon
1652		*salA*	lantibiotic salivaricin A precursor
1660		*lacR.2*	putative lactose phosphotransferase system repressor protein
1720		*dppC*	putative dipeptide ABC transporter, permease protein
1722	sag	dppE*	putative dipeptide ABC transporter, ATP-binding protein
1733			putative two-component response regulator
1740		*prsA*	foldase protein PrsA
1742		speB*	pyrogenic exotoxin B
1744		*ropB*	putative transcription regulator
1773		*hutU*	urocanate hydratase
1775			formiminotetrahydrofolate cyclodeaminase
1779		*hutH*	histidine ammonia-lyase

When the gene donor was identified as another *Streptococcus* species, the name of the donor is documented in the “LGT” column. Locus tag refers to the MGAS315 genome (SpyM3). Genes marked with an asterisk have significant BLASTp matches to established pathogenic bacteria virulence factors within the Virulence Factors of Pathogenic Bacteria database. LGT column shows donor species for genes gained via lateral gene transfer as detected by AnGST (see Table 1 for species IDs). Gene gains that were not robust to the species tree variation are not shown.

### The role of prophages

Although slightly more than half (58.4%) of the gained genes were annotated as hypothetical proteins, the remaining 47 genes had functional annotations, with 14 showing significant BLASTp matches to established pathogenic bacteria virulence factors within the Virulence Factors of Pathogenic Bacteria database (VFDB; [35]). For nine of the 13 *S. pyogenes* strains (MGAS315, SF370, MGAS5005, MGAS6180, MGAS9429, MGAS10270, MGAS2096, MGAS10750, and NZ131), their annotations contained descriptive information for phage genes. This information allowed us to determine if any of the 133 gained genes were located within a phage for any of these nine strains. We determined that 52 (46.0%) of the gained genes were located within a phage in at least one of these strains (Table 4). Five of these genes were virulence factors, so at least one third of the gained virulence factors were phage associated. Of the five virulence factors, four were established *S. pyogenes* virulence factors. The first two (*speK* and *speA3*) (Table 3), were streptococcal pyrogenic exotoxins, which are associated with streptococcal toxic shock syndrome and scarlet fever [44], [45]. The next two were exoenzyme spreading factors (*hylP*.1 and *hylP*.3), which degrade the hyaluronic acid of connective tissue, aiding spread of the pathogen [46]. The fifth gene, a putative single-strand DNA-binding protein, has significant sequence similarity to a *Salmonella enterica* ssDNA-binding protein involved in the regulation of recombination. In addition to the established virulence factors, there was also a putative ATP-binding cassette (ABC) transporter protein, which has been shown to be important in the virulence of other streptococcal bacteria [47], [48].

**Table 4:** Distribution of 52 phage genes gained on the *S. pyogenes* branch for nine strains that possessed phage descriptions in their annotations.

Gene ID	Strain ID								
	315	SF370	5005	6180	9429	10270	2096	10750	NZ131
1264*	315.6		5005.3	6180.2	9428.3	10270.3	2096.2		NZ131.2
1270*	315.2	370.2	5005.2		9429.1	10270.3		10750.3	NZ131.3
1586*	315.3	370.2			9429.2			10750.3	
1587*	315.5			6180.1	9429.1	10270.2		10750.2	
1590*	315.5			6180.1	9429.1	10270.2		10750.2	
1687*	315.4	370.1		6180.2		10270.3	2096.1		
1710*	315.3	370.2	5005.2		9429.2			10750.3	NZ131.2
1792*	315.6		5005.2	6180.2		10270.3			NZ131.2
1818*	315.4		5005.1	6180.2		10270.3			NZ131.3
1862*	315.4	370.1		6180.2		10270.3	2096.1		
1863*	315.4	370.1		6180.2		10270.3	2096.1		
1864*	315.4	370.1		6180.2		10270.3	2096.1		
1865*	315.4	370.1		6180.2		10270.3	2096.1		
1867*	315.4	370.1		6180.2		10270.3	2096.1		
1875*	315.1	370.3		6180.1	9429.2				NZ131.3
1906*	315.1	370.3		6180.1	9429.2				NZ131.3
1915*	315.4		5005.1	6180.2		10270.3			NZ131.3
1939*	315.3	370.2	5005.2					10750.3	
1957*	315.2				9429.1	10270.1		10750.1	
1969*	315.4			6180.2		10270.3			NZ131.3
1970*	315.4			6180.2		10270.3			NZ131.3
1975*			5005.3		9428.3		2096.2		NZ131.2
1977*	315.6		5005.3		9428.3		2096.2		NZ131.2
1993*	315.4	370.1		6180.2		10270.3	2096.1		
2094	315.3	370.2	5005.2					10750.3	
2121*	315.1								NZ131.3
2160*	315.5		5005.1	6180.1					
2191*	315.4			6180.2		10270.3			
2234*	315.6		5005.3		9428.3		2096.2		
2260*	315.5	370.1				10270.2	2096.1	10750.2	
2266*	315.2	370.3	5005.2	6180.1	9429.2				
2276*	315.6			6180.2		10270.3			
2297	315.4			6180.2		10270.3		10750.2	
2441*	315.5				9429.1	10270.1		10750.1	
2621			5005.1						
2667	315.2					10270.2		10750.2	NZ131.3
2722	315.3	370.2						10750.3	
2730	315.2					10270.1		10750.1	
2731	315.2				9429.1	10270.1		10750.1	
2733*			5005.1			10270.2		10750.2	
2735	315.4			6180.2		10270.3			
2736	315.4			6180.2		10270.3			
2745	315.2		5005.1	6180.1					NZ131.3
3051	315.5					10270.2		10750.2	
3069	315.5					10270.2		10750.2	
3070	315.4			6180.2		10270.3			
3071	315.4			6180.2		10270.3			
3072	315.4			6180.2		10270.3			
3080	315.3							10750.3	
3081	315.3							10750.3	
3087	315.2			6180.1	9429.1				
3088	315.4			6180.2					

Columns under strain IDs contain phage IDs. Asterisks mark genes that experienced LGT subsequent to being gained on the *S. pyogenes* branch. The strain ID prefix MGAS was omitted to save space.

**Figure 4:**
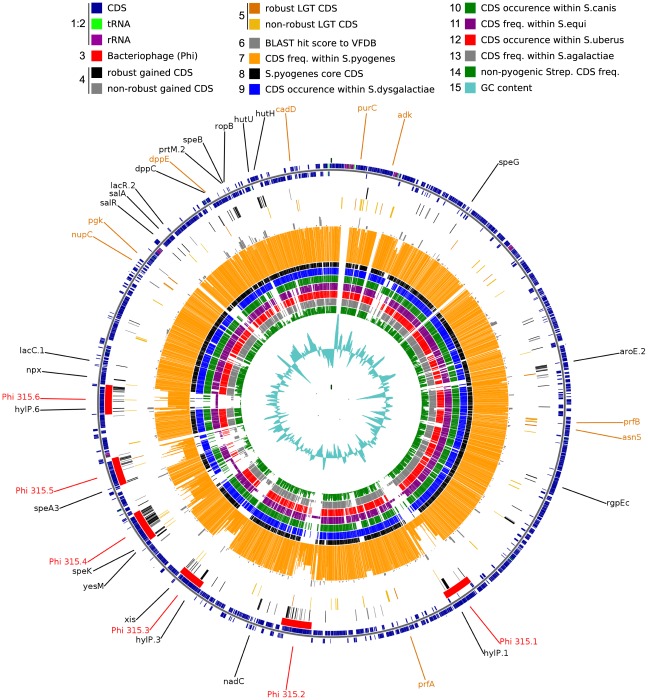
Circular map of the S. *pyogenes* MGAS315 genome showing location of gained and LGT genes. A color legend for each ring (1 to 15) appears at the top of the figure. The figure highlights the *S. pyogenes* core-genome distribution (tracks 7 and 8) and islands of gained genes (tracks 3 and 4). MGAS315 is chosen simply as a reference genome to map the results of the analysis.

AnGST analysis of evolutionary events subsequent to the origin of the species *S. pyogenes* indicated that of the 52 gained genes associated with phages (Fig. 4), 33 were involved in 90 subsequent LGT events, during the diversification of the different strains (Table 4). Over half of these events (46) were restricted to LGT among *S. pyogenes* strains, possibly reflecting the narrow host range of *S. pyogenes*, once it was strictly adapted to humans. Almost half (21) of the remaining 44 LGTs involved *Streptococcus equi* subsp. *equi*. This was followed by *Streptococcus agalactiae* (8), with *S. dysgalactiae* subsp. *equisimilis*, *S. canis*, *Streptococcus gallolyticus*, and *Streptococcus mitis* accounting for the remainder. This suggests a close association between *S. pyogenes* and *Streptococcus equi* subsp. *equi* and to a lesser extent *S. pyogenes* and *S. agalactiae*, with the former confirming the findings of Holden *et al.* [49], which showed that phages within *S. pyogenes* and *Streptococcus equi* subsp. *equi* were closely related, and the species shared “a common phage pool”. Intriguingly, both *S. pyogenes* and *S. equi* subsp. *equi* are host restricted (*S. equi* subsp. *equi* is restricted to horses where it is the causative agent of equine strangles). The high frequency of phage mediated LGT between these two species may reflect a close human-horse association and/or that *S. pyogenes* was an important factor in the evolution of *S. equi* subsp. *equi* as it split from *S. equi* subsp. *zooepidemicus* to become a strict horse pathogen [49]. The direction of phage mediated LGT between *S. pyogenes* and *S. equi* subsp. *equi* lends support to the latter hypothesis, as 85.7% of the LGTs between these two species were from *S. pyogenes* to *S. equi* subsp. *equi*. The taxon *S. equi*. subsp. *equi* must be of relatively recent age, since the clone is very homogeneous, with sequence divergence of housekeeping loci across diverse collections of strains extremely minimal [50] and microarray data of ours confirming this across the genome, while concomitantly indicating that relatively few genes comprise the dispensable component of the genome compared to other *Streptococcus* taxa ([14] and Stanhope unpublished data). *S. pyogenes* on the other hand, is clearly of much older origin. We suggest that phage mediated LGTs from *S. pyogenes* to one or a few *S. equi* subsp. *zooepidemicus* strains were instrumental in creating the progenitor or founder of *S. equi* subsp. *equi*, which then developed into the current version of this clonal organism. Such a scenario could be a rare example of reverse zoonosis, although it is also possible that this transpired within the human host, involving an instance of co-infection involving *S. pyogenes* and *S. equi* subsp. *zooepidemicus*. Cases of human infection by *S. equi* subsp. *zooepidemicus*, although not common, are nonetheless reported, involving both zoonotic transmission from domesticated animals [51], and the consumption of inadequately pasteurized milk products [52]. The genes involved in this *pyogenes*-*equi* LGT included hyaluronoglucosaminidase (*hylP*) and streptococcal pyrogenic exotoxin (*speK*) with the remainder annotated as hypotheticals or phage associated proteins. The majority (more than 75%) of these LGTs originated from serotype 5 and 49 strains. This is not to say, that we found no evidence of the reverse directionality in LGT – from *equi* to *pyogenes* – AnGST simply identified the majority of the LGT between these two taxa in the *pyogenes*-*equi* direction (17 vs 3).

### M-protein island

The M-protein pathogenicity island is a region of 35 genes present in all sequenced *S. pyogenes* genomes [53]. Three genes showing homology to the VFDB were located within this island: *dppE*, *speB*, and a putative two-component response regulator. *DppE* (gained via LGT from *S. agalactiae*) is one of six ABC transporter proteins present in the island, and *speB* is another streptococcal pyrogenic exotoxin. In addition to these three genes, the island contained five additional loci gained along the *S. pyogenes* branch (Table 3). One of these, *ropB* (also known as *rgg*) is of particular interest, due to its interaction with *speB*. Both *rgg* and *speB* have recently been shown to have greater expression in pharyngal conditions as opposed to invasive conditions [54]. Rgg regulates the expression of several virulence factors (including *speB*), as well as activates the utilization of non-glucose carbohydrates [55]. Transcriptome analyses have shown that *rgg* is over-expressed in saliva conditions [56] and in the adherence phase [57]. *SpeB* leads to the cleavage or inactivation of many bacterial proteins, including virulence factors involved in invasive disease that contribute to host-pathogen interaction [54]. Differential expression of *speB* may lead to different levels of lethality, because decreased production of *speB* results in the preservation of *S. pyogenes* virulence factors. Thus, both genes are playing an essential role for survival in human saliva. *SpeB*-*rgg* interaction may have contributed to *S. pyogenes* colonization of the human pharynx as its main habitat, without generating invasive disease that would kill the host and thereby reduce possibilities of dispersion. In that sense, the gain of *speB* and *rgg* might have been a critical component in *S. pyogenes* adaptation to the human host.

The M-protein, a key *S. pyogenes* virulence factor [16], was not gained along the *S. pyogenes* branch and is shared with several *Streptococcus* species; for example, *S. dysgalactiae* subsp. *equi*similis, *S. dysgalactiae* subsp. *dysgalactiae*, and *S. agalactiae* [58]. Indeed, a large proportion of the M-protein island genes are shared with other *Streptococcus* species [58]. However, it is only in *S. pyogenes* that the genes form a contiguous island [53], [58]. Consequently, it appears that while the majority (77%) of the M-protein island genes were present in the *S. pyogenes* ancestor, it was here that they clustered to form a contiguous island, highlighting the importance of gene rearrangement in addition to gene gain as an important evolutionary factor in the emergence of new species.

### The role of *Streptococcus agalactiae*

*Streptococcus agalactiae* appears to have played an important role in the evolution of *S. pyogenes* with 10 of the 14 LGTs having a *Streptococcus* donor, originating from this species (Table 3). Three of these laterally transferred genes (*prfB*, *asnC*, and an acetoin reductase) are clustered together within *S. pyogenes* genome sequences. Two of these genes (*asnC* and acetoin reductase) showed significant BLASTp matches to *Escherichia coli* virulence factors. *asnC* was similar to *LysU*, a heat shock inducible lysyl-rRNA synthase that enables pathogen survival at elevated temperatures [59]. Acetoin reductase was similar to *entA*, which is involved in the biosynthesis and excretion of the siderophore enterobactin that enables survival in iron poor environments such as the urinary tract [60], [61]. Comparison to *S. agalactiae* (NEM316) showed these three genes to be contained within an approximately 11 kbp region that shared approximately 76% sequence identity with *S. pyogenes*, suggesting that this entire region may have been historically involved in LGT with *S. agalactiae*.

Examination of a recently identified *S. pyogenes* virulence factor, the *sal* lantibiotic locus, provides further evidence supporting LGT between *S. pyogenes* and *S. agalactiae*. First reported in *Streptococcus salivarius*, the *sal* locus contains seven genes (*salAMTXYKR*) and is involved in production and immunity to the lantibiotic salivaricin A [62]. Subsequently, the locus has been reported in both *S. agalactiae* and *S. pyogenes* [33], [63]. However, in *S. agalactiae*, production and immunity to salavaricin A is rare and restricted to isolates from the bovine host [33], [63]; whereas in *S. pyogenes*, nearly all strains tested, lack immunity to salivaricin A [62], [63], [64]. Assuming that production and immunity to salivaricin A is the ancestral state for this locus, the locus appears to be functionally more derived in both *S. agalactiae* and *S. pyogenes*. Indeed, the *salM* and *salT* genes are truncated or disrupted in many *S. pyogenes* strains [63]. Similarly, the *salM* gene is truncated and the *salA* gene missing in bovine adapted *S. agalactiae* (strain FSL S3-026) [33]. Phelps and Neely [65] demonstrated that for *S. pyogenes*, the locus had shifted its immunity function from salivaricin to the host immune system, with the *salY* gene of the locus now required for survival within macrophages. Furthermore, alignment of *S. pyogenes* (strain MGAS315) (all genes of the locus are intact in this strain) to the bovine *S. agalactiae* strain, and *S. salivarius* (strain 20P3), showed very high sequence identity (97.6%) between *S. pyogenes* and *S. agalactiae*, but somewhat lower identity between these two species and *S. salivarius* (92.9% and 93.7% respectively).

Given that the lantibiotic operon of *S. pyogenes* is adapted to an alternate function that allows the bacteria to colonize intra-cellular environments, and in particular, provides it the possibility to survive within macrophages, this may have had an important impact on the success of *S. pyogenes*, not only because it makes *S. pyogenes* resistant to phagocytosis, but also because phagocytic cells may serve as a reservoir of infection and a refuge to antibiotic treatment [66], as well as facilitating asymptomatic carriage of *S. pyogenes* [67]. The gain and adaptation of this sal locus has probably allowed *S. pyogenes* to colonize new and sterile tissues, which in turn are conditions that could trigger an adaptive habitat shift, accelerating the differentiation of *S. pyogenes*.

### Isolated genes

Several genes gained on the *S. pyogenes* branch not contained within phages or gene clusters are also implicated in virulence. For example, in addition to the three pyrogenic exotoxin genes already mentioned, a fourth (*speG*) was also gained on the *S. pyogenes* branch. This gene, along with speK, was involved in subsequent LGT events after the origin of the species *S. pyogenes*. *SpeG* was transferred to *S. dysgalactiae* subsp. *equisimilis* and *speK* was transferred to *S. equi* subsp. *equi*. Other genes gained on the *S. pyogenes* branch that showed significant sequence similarity to established virulence factors were *yesM* and *purC*. *YesM* shows similarity to *algZ* of *Pseudomonas aeruginosa*, which has been implicated in alginate (mucoid) production in *P. aeruginosa* strains in cystic fibrosis patients [68], whereas *purC* has similarity to *purC* of *Mycobacterium tuberculosis*, and disruption of this gene has been shown to attenuate the ability of *M. tuberculosis* and *Mycobacterium bovis* to multiply within mouse bone marrow macrophages [69].

### Conclusion

The emergence of *S. pyogenes* was ultimately linked to its strict adaptation to the human host and particularly to the human saliva and pharynx environment. We have shown that adaptation to this new habitat was achieved in part by (i) the integration of new virulence factors (e.g. *speB*, and the *sal* locus) and (ii) the construction of new regulation networks (e.g. *rgg*, and to some extent *speB*). While the virulence factors were undoubtedly important in allowing *S. pyogenes* to survive and compete with human host defenses, it is also apparent that the regulation of newly acquired or already existing virulence factors was a fundamental issue. Two recent studies have shown that a single frameshift mutation in a regulatory gene (*covRS*), causes *S. pyogenes* to switch from a local to an invasive infection [54], [70]. This shift is due to the transition to a fundamentally different transcriptome [54], where the expression of *speB* is abolished, preventing the degradation of other virulence factors and allowing them to reach host tissues [70]. An extreme invasive disease phenotype would be an evolutionary dead end for any pathogen, as it would kill the host and reduce success of dispersal and colonization of new hosts. Thus, it would appear that during the evolution of *S. pyogenes*, new regulation networks (e.g. *rgg*) were integrated with already existing ones (e.g. the *covRS*), providing a more sensitive global regulation network, which in turn was instrumental in the adaptation of the species as a long term strict human pathogen. This highlights the fundamental role of regulation in the host/pathogen relationship, and suggests the need for further comparative analysis that would integrate non-coding functional elements and their role in virulence regulation (e.g. [71])

## References

[1] Musser JM, Hauser AR, Kim MH, Schlievert PM, Nelson K (1991). *Streptococcus pyogenes* causing toxic-shock-like syndrome and other invasive diseases: clonal diversity and pyrogenic exotoxin expression.. Proc Natl Acad Sci U S A.

[2] Smoot JC, Barbian KD, Van Gompel JJ, Smoot LM, Chaussee MS (2002). Genome sequence and comparative microarray analysis of serotype M18 group A *Streptococcus* strains associated with acute rheumatic fever outbreaks.. Proc Natl Acad Sci U S A.

[3] Colman G, Tanna A, Efstratiou A, Gaworzewska ET (1993). The serotypes of *Streptococcus pyogenes* present in Britain during 1980–1990 and their association with disease.. J Med Microbiol.

[4] Beres SB, Richter EW, Nagiec MJ, Sumby P, Porcella SF (2006). Molecular genetic anatomy of inter- and intraserotype variation in the human bacterial pathogen group A Streptococcus.. Proc Natl Acad Sci U S A.

[5] Ferretti JJ, McShan WM, Ajdic D, Savic DJ, Savic G (2001). Complete genome sequence of an M1 strain of *Streptococcus pyogenes*.. Proc Natl Acad Sci U S A.

[6] Sumby P, Porcella SF, Madrigal AG, Barbian KD, Virtaneva K (2005). Evolutionary origin and emergence of a highly successful clone of serotype M1 group a *Streptococcus* involved multiple horizontal gene transfer events.. The Journal of Infectious Diseases.

[7] Beres SB, Sylva GL, Barbian KD, Lei B, Hoff JS (2002). Genome sequence of a serotype M3 strain of group A *Streptococcus*: phage-encoded toxins, the high-virulence phenotype, and clone emergence.. Proc Natl Acad Sci U S A.

[8] Green NM, Zhang S, Porcella SF, Nagiec MJ, Barbian KD (2005). Genome sequence of a serotype M28 strain of group a *Streptococcus*: potential new insights into puerperal sepsis and bacterial disease specificity.. The Journal of Infectious Diseases.

[9] Nakagawa I, Kurokawa K, Yamashita A, Nakata M, Tomiyasu Y (2003). Genome sequence of an M3 strain of *Streptococcus pyogenes* reveals a large-scale genomic rearrangement in invasive strains and new insights into phage evolution.. Genome Res.

[10] Banks DJ, Porcella SF, Barbian KD, Beres SB, Philips LE (2004). Progress toward characterization of the group A *Streptococcus* metagenome: complete genome sequence of a macrolide-resistant serotype M6 strain.. The Journal of Infectious Diseases.

[11] Holden MT, Scott A, Cherevach I, Chillingworth T, Churcher C (2007). Complete genome of acute rheumatic fever-associated serotype M5 *Streptococcus* pyogenes strain manfredo.. J Bacteriol.

[12] McShan WM, Ferretti JJ, Karasawa T, Suvorov AN, Lin S (2008). Genome sequence of a nephritogenic and highly transformable M49 strain of *Streptococcus pyogenes*.. J Bacteriol.

[13] McCormick JK, Peterson ML, Schlievert PM (2006). Toxins and superantigens of group A streptococci. Gram-positive pathogens.

[14] Lefebure T, Stanhope MJ (2007). Evolution of the core and pan-genome of *Streptococcus*: positive selection, recombination, and genome composition.. Genome Biol.

[15] Musser JM, DeLeo FR (2005). Toward a genome-wide systems biology analysis of host-pathogen interactions in group A *Streptococcus*.. The American Journal of Pathology.

[16] Cunningham MW (2000). Pathogenesis of group A streptococcal infections.. Clin Microbiol Rev.

[17] Aziz RK, Edwards RA, Taylor WW, Low DE, McGeer A (2005). Mosaic prophages with horizontally acquired genes account for the emergence and diversification of the globally disseminated M1T1 clone of *Streptococcus pyogenes*.. J Bacteriol.

[18] Beres SB, Musser JM (2007). Contribution of exogenous genetic elements to the group A *Streptococcus* metagenome.. PLoS One.

[19] Tapp J, Thollesson M, Herrmann B (2003). Phylogenetic relationships and genotyping of the genus *Streptococcus* by sequence determination of the RNase P RNA gene, rnpB.. Int J Syst Evol Microbiol.

[20] Jensen A, Kilian M (2012). Delineation of *Streptococcus dysgalactiae*, Its Subspecies, and Its Clinical and Phylogenetic Relationship to *Streptococcus pyogenes*.. J Clin Microbiol.

[21] Lam MM, Clarridge JE, Young EJ, Mizuki S (2007). The other group G *Streptococcus*: increased detection of *Streptococcus canis* ulcer infections in dog owners.. J Clin Microbiol.

[22] Galperine T, Cazorla C, Blanchard E, Boineau F, Ragnaud JM (2007). *Streptococcus canis* infections in humans: retrospective study of 54 patients.. J Infect.

[23] Tikofsky LL, Zadoks RN (2005). Cross-infection between cats and cows: origin and control of *Streptococcus canis* mastitis in a dairy herd.. J Dairy Sci.

[24] Murase T, Morita T, Sunagawa Y, Sawada M, Shimada A (2003). Isolation of *Streptococcus canis* from a Japanese raccoon dog with fibrinous pleuropneumonia.. Vet Rec.

[25] DeWinter LM, Prescott JF (1999). Relatedness of *Streptococcus canis* from canine streptococcal toxic shock syndrome and necrotizing fasciitis.. Can J Vet Res.

[26] Sykes JE, Kittleson MD, Pesavento PA, Byrne BA, MacDonald KA (2006). Evaluation of the relationship between causative organisms and clinical characteristics of infective endocarditis in dogs: 71 cases (1992–2005).. J Am Vet Med Assoc.

[27] Rolston KV (1986). Group G streptococcal infections.. Arch Intern Med.

[28] Brandt CM, Spellerberg B (2009). Human infections due to *Streptococcus dysgalactiae* subspecies *equisimilis*.. Clin Infect Dis.

[29] Margulies M, Egholm M, Altman WE, Attiya S, Bader JS (2005). Genome sequencing in microfabricated high-density picolitre reactors.. Nature.

[30] Zerbino DR, Birney E (2008). Velvet: algorithms for *de novo* short read assembly using de Bruijn graphs.. Genome Res.

[31] Li L, Stoeckert C, Roos DS (2003). OrthoMCL: identification of ortholog groups for eukaryotic genomes.. Genome Res.

[32] Lefebure T, Bitar PD, Suzuki H, Stanhope MJ (2010). Evolutionary dynamics of complete *Campylobacter* pan-genomes and the bacterial species concept.. Genome Biol Evol.

[33] Richards VP, Lang P, Bitar PD, Lefebure T, Schukken YH (2011). Comparative genomics and the role of lateral gene transfer in the evolution of bovine adapted *Streptococcus agalactiae*.. Infect Genet Evol.

[34] Enright AJ, Van Dongen S, Ouzounis CA (2002). An efficient algorithm for large-scale detection of protein families.. Nucleic Acids Res.

[35] Yang J, Chen L, Sun L, Yu J, Jin Q (2008). VFDB 2008 release: an enhanced web-based resource for comparative pathogenomics.. Nucleic Acids Res.

[36] Lefebure T, Stanhope MJ (2009). Pervasive, genome-wide positive selection leading to functional divergence in the bacterial genus *Campylobacter*.. Genome Res.

[37] Roshan U, Livesay DR (2006). Probalign: multiple sequence alignment using partition function posterior probabilities.. Bioinformatics.

[38] Guindon S, Dufayard JF, Lefort V, Anisimova M, Hordijk W (2010). New algorithms and methods to estimate maximum-likelihood phylogenies: assessing the performance of PhyML 3.0.. Syst Biol.

[39] Ane C, Larget B, Baum DA, Smith SD, Rokas A (2007). Bayesian estimation of concordance among gene trees.. Mol Biol Evol.

[40] Ronquist F, Huelsenbeck JP (2003). MrBayes 3: Bayesian phylogenetic inference under mixed models.. Bioinformatics.

[41] David LA, Alm EJ (2011). Rapid evolutionary innovation during an Archaean genetic expansion.. Nature.

[42] Maddison W (1997). Gene Trees in Species Trees.. Systematic Biology.

[43] Hudson RR (1992). Gene trees, species trees and the segregation of ancestral alleles.. Genetics.

[44] Talkington DF, Schwartz B, Black CM, Todd JK, Elliott J (1993). Association of phenotypic and genotypic characteristics of invasive *Streptococcus pyogenes* isolates with clinical components of streptococcal toxic shock syndrome.. Infect Immun.

[45] Muller-Alouf H, Carnoy C, Simonet M, Alouf JE (2001). Superantigen bacterial toxins: state of the art.. Toxicon.

[46] Starr CR, Engleberg NC (2006). Role of hyaluronidase in subcutaneous spread and growth of group A streptococcus.. Infect Immun.

[47] Basavanna S, Khandavilli S, Yuste J, Cohen JM, Hosie AH (2009). Screening of *Streptococcus pneumoniae* ABC transporter mutants demonstrates that LivJHMGF, a branched-chain amino acid ABC transporter, is necessary for disease pathogenesis.. Infect Immun.

[48] Jones AL, Knoll KM, Rubens CE (2000). Identification of *Streptococcus agalactiae* virulence genes in the neonatal rat sepsis model using signature-tagged mutagenesis.. Mol Microbiol.

[49] Holden MT, Heather Z, Paillot R, Steward KF, Webb K (2009). Genomic evidence for the evolution of *Streptococcus equi*: host restriction, increased virulence, and genetic exchange with human pathogens.. PLoS Pathog.

[50] Webb K, Jolley KA, Mitchell Z, Robinson C, Newton JR (2008). Development of an unambiguous and discriminatory multilocus sequence typing scheme for the *Streptococcus zooepidemicus* group.. Microbiology.

[51] Eyre DW, Kenkre JS, Bowler IC, McBride SJ (2010). *Streptococcus equi* subspecies zooepidemicus meningitis–a case report and review of the literature.. Eur J Clin Microbiol Infect Dis.

[52] Kuusi M, Lahti E, Virolainen A, Hatakka M, Vuento R (2006). An outbreak of *Streptococcus equi* subspecies zooepidemicus associated with consumption of fresh goat cheese.. BMC Infect Dis.

[53] Panchaud A, Guy L, Collyn F, Haenni M, Nakata M (2009). M-protein and other intrinsic virulence factors of *Streptococcus pyogenes* are encoded on an ancient pathogenicity island.. BMC Genomics.

[54] Sumby P, Whitney AR, Graviss EA, DeLeo FR, Musser JM (2006). Genome-wide analysis of group A streptococci reveals a mutation that modulates global phenotype and disease specificity.. PLoS Pathogens.

[55] Dmitriev AV, McDowell EJ, Kappeler KV, Chaussee MA, Rieck LD (2006). The Rgg regulator of *Streptococcus pyogenes* influences utilization of nonglucose carbohydrates, prophage induction, and expression of the NAD-glycohydrolase virulence operon.. J Bacteriol.

[56] Shelburne SA, Granville C, Tokuyama M, Sitkiewicz I, Patel P (2005). Growth characteristics of and virulence factor production by group A *Streptococcus* during cultivation in human saliva.. Infect Immun.

[57] Ryan PA, Kirk BW, Euler CW, Schuch R, Fischetti VA (2007). Novel algorithms reveal streptococcal transcriptomes and clues about undefined genes.. PLoS Computational Biology.

[58] Suzuki H, Lefebure T, Hubisz MJ, Pavinski Bitar P, Lang P (2011). Comparative genomic analysis of the *Streptococcus dysgalactiae* species group: gene content, molecular adaptation, and promoter evolution.. Genome Biol Evol.

[59] Rudolph B, Gebendorfer KM, Buchner J, Winter J (2010). Evolution of *Escherichia coli* for growth at high temperatures.. J Biol Chem.

[60] Khalil S, Pawelek PD (2011). Enzymatic adenylation of 2,3-dihydroxybenzoate is enhanced by a protein-protein interaction between *Escherichia coli* 2,3-dihydro-2,3-dihydroxybenzoate dehydrogenase (EntA) and 2,3-dihydroxybenzoate-AMP ligase (EntE).. Biochemistry (Mosc).

[61] Wiles TJ, Kulesus RR, Mulvey MA (2008). Origins and virulence mechanisms of uropathogenic *Escherichia coli*.. Exp Mol Pathol.

[62] Ross KF, Ronson CW, Tagg JR (1993). Isolation and characterization of the lantibiotic salivaricin A and its structural gene salA from *Streptococcus salivarius* 20P3.. Appl Environ Microbiol.

[63] Wescombe PA, Upton M, Dierksen KP, Ragland NL, Sivabalan S (2006). Production of the lantibiotic salivaricin A and its variants by oral streptococci and use of a specific induction assay to detect their presence in human saliva.. Appl Environ Microbiol.

[64] Simpson WJ, Ragland NL, Ronson CW, Tagg JR (1995). A lantibiotic gene family widely distributed in *Streptococcus salivarius* and *Streptococcus pyogenes*.. Dev Biol Stand.

[65] Phelps HA, Neely MN (2007). SalY of the *Streptococcus pyogenes* lantibiotic locus is required for full virulence and intracellular survival in macrophages.. Infect Immun.

[66] Thulin P, Johansson L, Low DE, Gan BS, Kotb M (2006). Viable group A streptococci in macrophages during acute soft tissue infection.. PLoS Medicine.

[67] Tart AH, Walker MJ, Musser JM (2007). New understanding of the group A *Streptococcus* pathogenesis cycle.. Trends Microbiol.

[68] Wozniak DJ, Sprinkle AB, Baynham PJ (2003). Control of *Pseudomonas aeruginosa* algZ expression by the alternative sigma factor AlgT.. J Bacteriol.

[69] Jackson M, Phalen SW, Lagranderie M, Ensergueix D, Chavarot P (1999). Persistence and protective efficacy of a *Mycobacterium tuberculosis* auxotroph vaccine.. Infect Immun.

[70] Walker MJ, Hollands A, Sanderson-Smith ML, Cole JN, Kirk JK (2007). DNase Sda1 provides selection pressure for a switch to invasive group A streptococcal infection.. Nat Med.

[71] Bejerano G, Siepel AC, Kent WJ, Haussler D (2005). Computational screening of conserved genomic DNA in search of functional noncoding elements.. Nature Methods.

